# Comparison of topical treatment methods used in recurrent anterior epistaxis: a randomized clinical trial

**DOI:** 10.1016/j.bjorl.2019.07.002

**Published:** 2019-08-11

**Authors:** Hasan Emre Koçak, Zeki Tolga Bilece, Mehmet Keskin, Hüseyin Avni Ulusoy, Arzu Karaman Koç, Kamil Hakan Kaya

**Affiliations:** Bakırköy Dr. Sadi Konuk Training and Research Hospital, Department of Otorhinolaryngology, Head and Neck Surgery, Istanbul, Turkey

**Keywords:** Epistaxis, Treatment, Antiseptic, Decongestant, Chemical cauterization

## Abstract

**Introduction:**

Recurrent epistaxis is a common medical problem faced by ENT specialists, emergency physicians, and pediatricians. The facts that many treatment modalities are being searched and no single treatment method is universally accepted yet support this information.

**Objective:**

We aimed to compare the clinical efficacy of topical antiseptic ointment, topical decongestant ointment and chemical cauterization treatments, which are frequently used in recurrent anterior epistaxis, both singly and in combination.

**Material-methods:**

Between August 2017 and February 2018, 137 patients who were diagnosed with recurrent anterior epistaxis were randomly divided into 5 groups. group I received topical antiseptic ointment, group II received topical decongestant ointment, group III received chemical cauterization, group IV received topical antiseptic ointment + chemical cauterization and group V received topical decongestant ointment + chemical cauterization treatment. All patients were phoned 2 weeks and 1 month after the treatment and questioned about the presence (failure) or absence (success) of at least 1 episode of epistaxis. Patients with comorbid diseases were excluded. Treatment success was statistically analysed.

**Results:**

There was no significant difference (*p* > 0.05) between the groups in the success rate at 15th day after treatment. Group IV and group V had higher success rates at 30th day after treatment compared with group I and group II (*p* < 0.05). In group III 30th day treatment success was not different from the other 4 groups (*p* > 0.05).

**Conclusion:**

Although the number of patients who improved with chemical cauterization (group III) was higher in our study, no significant difference was observed in single treatment modalities (group I‒III) at 14th day and 30th day after treatment. Although no statistically significant difference was observed between combined treatments (group IV—V) and single treatments (group I‒III) in the 2nd week after treatment, combined treatments were significantly more effective in the 1st month.

## Introduction

**Figure 1:**
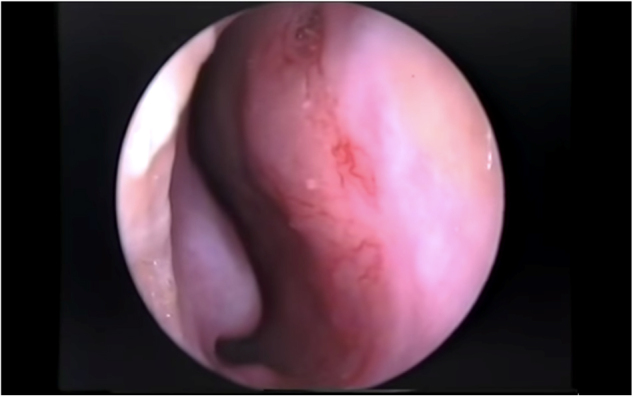
The little area (Kiesselbach's plexus): anterior to the nasal septum.

Epistaxis, which is bleeding from the nose, is seen in 60% of the population. Eighty percent of these cases arise from the Little’s area (Kiesselbach's plexus) anterior to the nasal septum and are called anterior epistaxis (Fig. 1).^1^ It is a common problem for the Ear-Nose-Throat (ENT) specialist, paediatrician and emergency physician.^2^ Anterior epistaxis originating from Little’s area could be repetitive, develops spontaneously, can be usually stopped easily by applying digital pressure on nose sidewalls, and is usually not life-threatening but it causes great anxiety in patients and their relatives.^3^ Recurrent epistaxis is usually associated with crusting, nasal vestibulitis, rhinosinusitis, digital trauma (sniffling, nose-picking) but generally a direct cause can't be found.^4^ Therefore, topical ointments that include oil and decongestants and that moisturize nasal cavities have a role in treatment.^5^ Nasal colonization with *Staphylococcus aureus* is also thought to play a role and therefore treatment with an antiseptic ointment could be also important.^6^ Treatment of vascular telangiectasia detected by anterior rhinoscopic examination by chemical cauterization is an alternative treatment method that can be applied in clinical settings.^7^ It is a fact that no single treatment method has been accepted universally for the treatment of recurrent epistaxis.^8^ Our purpose in this study was to compare the clinical effectiveness of these frequently applied treatments for recurrent anterior epistaxis both singly and in combination.

## Methods

### Ethics committee

This study was performed after local ethics committee approval (Ethics Committee Number: 2017-230) as a prospective randomized clinical trial.

### Patient selection

This study included 137 patients admitted to our clinic with recurrent epistaxis, detected to have localized vascular telangiectasias at the Little’s area in anterior rhinoscopic evaluation, and diagnosed as recurrent anterior epistaxis between August 2017 and February 2018. Patients detected by anterior rhinoscopic examination of the nasal septum to have telangiectasia that were thought to be causative of hemorrhage were randomly grouped to test different treatments.

### Treatment planning

Local antiseptic ointment including oxytetracycline hydrochloride-polymyxin B sulphate (Terramycin, Pfizer Corp. Istanbul, Turkey) and local decongestant ointment including ephedrine-naphazoline (Sulfarhin-Santa Farma Corp. Istanbul, Turkey) were given to the patients for intranasal topical treatment. The patients were instructed to apply the ointment to the nostril on the lesion side twice a day for 2 week and then compress the nostril.

Chemical cauterization was performed using silver nitrate sticks. Local anaesthesia before the procedure was achieved by applying a cotton tamponade soaked with 2 ml 10% lidocaine. (Xylocaine 10% Spray, Astra Zeneca Corp. Istanbul, Turkey). After 10 min, the cauterizing side of a silver nitrate stick (75% silver nitrate, 25% potassium nitrate, Selam Medical Corp., Istanbul, Turkey) was applied for a few seconds to the telangiectasia area until a white precipitate is formed. Caution was exercised to prevent damage by cauterization to the surrounding healthy nasal mucosa.

### Groupage

The patients were grouped five groups by simple randomization. In the context of the study criteria, patients were assigned to the groups with the help of a computer program completely randomly, equally and independently, regardless of the previous participation. Group I received topical antiseptic ointment, group II received topical decongestant ointment, group III received chemical cauterization, group IV received topical antiseptic ointment + chemical cauterization and group V received topical decongestant ointment + chemical cauterization. All patients were phoned 2 weeks and 1 month after the treatment and questioned about the presence (failure) or absence (success) of at least one episode of epistaxis. Only one physician made phone calls and did not know which groups the patients belonged to.

### Inclusion criteria

Patients complaining of a single-sided nasal bleeding that recurred at least 4 times in the last month, who had localized telangiectasias in the Little’s area in anterior rhinoscopic evaluation and who accepted treatment were included.

### Exclusion criteria

Smokers and patients who presented comorbid diseases such as chronic cardiovascular, microvascular disease, chronic primary hypertension, diabetes mellitus Type 1 or 2, chronic obstructive pulmonary disease, asthma, hyperlipidemia, hypercholesterolemia, obesity, metabolic syndrome, using drugs users of coagulopathy-causing drugs, patients with bleeding disorders, acute and chronic rhinosinusitis were excluded.

### Statistical methods

Mean, standard deviation, median, lowest, highest, frequency and ratio values ​​were used as the descriptive statistics of the data. Distributions of the variables were assessed with the Kolmogorov–Smirnov test. Kruskal–Wallis test was used in the analysis of independent quantitative data. Chi-Square test was used to analyse independent qualitative data. SPSS 22.0 program was used to perform the statistical analyses.

## Results

The mean age of the patients participating in the study was 18.6 ± 14.9 (Table 1). The mean age of the groups was 18.1 ± 12.4, 20.2 ± 19.9, 18.0 ± 12.0, 20.2 ± 15.4, and 16.5 ± 14.6, respectively. No significant difference could be found among ages of the groups (*p* > 0.05) (Table 2). There were 45 male and 92 female patients in the study and the gender distribution did not differ significantly (*p* > 0.05) (Table 2). There was no significant difference (*p* > 0.05) between the treatment groups according to success at 15th day after the treatment (Table 2). Group IV and group V had higher success rates at 30th day after treatment compared with group I and group II (*p* < 0.05). In group III 30th day treatment success was not different from the other 4 groups (*p* > 0.05) (Table 2). There was no significant difference (*p* > 0.05) between group IV and group V treatment groups at 30th day treatment success (Table 2).

**Table 1:** Distribution of patients' age, gender and treatment success on the 15th and 30th day.

		Medyan	Mean ± SD/n (%)
Age		15.0	18.6 ± 14.9
Gender	Male		45 (32.8%)
	Female		92 (67.2%)
After 15 day	Unsuccessful		52 (38.0%)
	Successful		85 (62.0%)
After 30 day	Unsuccessful		27 (19.7%)
	Successful		110 (80.3%)

**Table 2:** Statistical analysis of the groups' age, gender and treatment successes at 15 and 30 days between groups.

	Group I		Group II		Group III		Group IV		Group V		*p*
	Mean ± S, n (%)	Med	Mean ± SD, n (%)	Med	Mean ± SD, n (%)	Med	Mean ± SD, n (%)	Med	Mean ± SD, n (%)	Med	
Age	18.1 ± 12.4	15.0	20.2 ± 19.9	12.0	18.0 ± 12.0	16.5	20.2 ± 15.4	16.5	16.5 ± 14.6	11.0	0.684 K
Gender											0.565 x²
Male	9 (34.6%)		10 (40.0%)		7 (26.9%)		12 (40.0%)		7 (23.3%)		
Female	17 (65.4%)		15(60.0%)		19 (73.1%)		18 (60.0%)		23 (76.7%)		
After 15 day											0.277 x²
Unsuccessful	13 (50.0%)		12 (48.0%)		10 (38.5%)		9 (30.0%)		8 (26.7%)		
Successful	13 (50.0%)		13 (52.0%)		16 (61.5%)		21 (70.0%)		22 (73.3%)		
After 30 day											0.026 x²
Unsuccessful	9 (34.6%)		8 (32.0%)		5 (19.2%)		2 (6.7%)		3 (10.0%)		
Successful	17 (65.4%)^a^, ^b^		17 (68.0%)^a^, ^b^		21 (80.8%)		28 (93.3%)		27 (90.0%)		

K, Kruskal-wallis; X², Chi-square test.

a Difference with Group IV, *p* < 0.05.

b Difference with Group V, *p* < 0.05.

## Discussion

Recurrent epistaxis is a common problem faced by ENT specialists, emergency physicians, and pediatricians. Direct examination (with or without a flexible or rigid endoscope) to exclude bleeding secondary to systemic disease usually reveals a vessel prone to bleeding at anterior nasal septum.^9^ We prospectively evaluated treatment strategies that were accepted to be effective in the literature singly or in combination by randomly forming treatment groups.

Treatment in epistaxis is somewhat easier because bleeding originates from the anterior septum in most of the cases.^10^ If there is no unusual condition such as coagulopathy, tumor, perforation, or hereditary hemorrhagic telangiectasia, bleeding usually originates from Kiesselbach's plexus. No special equipment is needed for diagnosis and treatment and most of the patients can be treated as outpatients unless profuse bleeding occurs.^11^ For this purpose, simple silver nitrate cautery has gained popularity among ENT specialists. However, its use requires a training and is not free of complications. Local anaesthesia is required for all cases and most local anaesthetics have adverse effects. Special caution should be exercised during bilateral septum cauterization due to the risk of septal perforation. Cauterization causes sclerosis in vessels and thickening of mucosa.^12^ The use of oils or ointments in the management of epistaxis has been previously suggested.^11^ Due to the hypothesis that septal mucosa in a specific region may be locally traumatized and result in recurrent bleeding, some authors suggest moisturization and oil and nasal serum physiologic drops to avoid excessive drying and crusting.^5^ It has been proven by some authors that the vasoconstriction that the topical decongestant application causes in Little’s area has a role in the treatment of epistaxis.^13^ Nasal colonization with *S. aureus* is also thought to play a role in the etiopathogenesis of epistaxis and the efficacy of treatment with this antiseptic pomade has been demonstrated in prospective randomized trials.^14^

We randomly created 5 groups in our study. The first three groups received antiseptic ointment, decongestant ointment, or silver nitrate which are used for management of epistaxis in clinical settings, while the other 2 groups received combinations of these treatments. Although treatment success rate was higher numerically in the silver nitrate group, no statistically significant difference could be observed in 14th day and 30th day treatment successes in single treatment modalities. The success of the combined treatments was not statistically significant at the 14th day but statistically significant at the 30th day.

There are several comparisons of treatment options for epistaxis in the literature. Ruddy et al. compared antiseptic treatment and cauterization and reported both treatments to be effective after 4 weeks of follow up in epistaxis treatment of 48 patients.^15^ Murthy et al. found same effectiveness for antiseptic treatment and cauterization in 8 week follow up of 64 epistaxis patients.^16^ On the contrary, Calder and colleagues found that combination of antiseptic cream and silver nitrate cautery was more effective than antiseptic cream alone in the treatment of 109 epistaxis cases with a 4 week follow-up.^17^ Kubba et al. compared untreated patients and those treated with antiseptic cream in their prospective randomized trial including 103 patients and reported a better result in the treatment group.^18^ Loughan et al. found in 105 epistaxis patients that vaseline alone was not useful.^19^ Topical antiseptic ointment, topical decongestant ointment and silver nitrate cautery are commonly used by ENT specialists in clinical settings. This is the first study in the literature that made prospective and randomized comparison of these treatments singly and in combination.

In our study, the cessation of bleeding episodes was considered as the only outcome parameter and the bleeding rate and amount were not considered. One reason for this preference is that the amount of bleeding self-reported by the patients is usually exaggerated and therefore unreliable. Complaints of nose bleeding which were repeated at least 4 times in the last month were taken into consideration as a criterion in questioning the amount of bleeding in the pre-treatment period. Although using cessation of bleeding as the only success criteria has decreased success rate in this study, it is an important outcome because it is the only factor that affects treatment.

## Conclusion

Local antiseptic ointment, local decongestant ointment, and chemical cauterization provide similar results for the treatment of epistaxis. Although no statistically significant difference was observed between combined treatments and single treatments in the 2nd week after treatment, combined treatments were significantly more effective in the 1st month.

## Ethical approval

All procedures performed in studies involving human participants were in accordance with the ethical standards of the institutional and/or national research committee and with the 1964 Helsinki declaration and its later amendments or comparable ethical standards.

## Conflicts of interest

The authors declare no conflicts of interest.
